# An Immortalized Hepatocyte-Like Cell Line (imHC) Accommodated Complete Viral Lifecycle, Viral Persistence Form, cccDNA and Eventual Spreading of a Clinically-Isolated HBV

**DOI:** 10.3390/v11100952

**Published:** 2019-10-16

**Authors:** Khanit Sa-ngiamsuntorn, Piyanoot Thongsri, Yongyut Pewkliang, Adisak Wongkajornsilp, Pattida Kongsomboonchoke, Phichaya Suthivanich, Suparerk Borwornpinyo, Suradej Hongeng

**Affiliations:** 1 Department of Biochemistry, Faculty of Pharmacy, Mahidol University, Bangkok 10400, Thailandpiyanoot20@hotmail.co.th (P.T.); 2 Excellent Center for Drug Discovery, Faculty of Science, Mahidol University, Bangkok 10400, Thailand; yongyut.pew@gmail.com (Y.P.); phichaya.sut@gmail.com (P.S.); bsuparerk@gmail.com (S.B.); 3 Department of Biotechnology, Faculty of Science, Mahidol University, Bangkok 10400, Thailand; gee.pattida@gmail.com; 4 Department of Pharmacology, Faculty of Medicine Siriraj Hospital, Mahidol University, Bangkok 10700, Thailand; 5 Department of Pediatrics, Faculty of Medicine Ramathibodi Hospital, Mahidol University, Bangkok 10400, Thailand

**Keywords:** hepatitis B, viral spreading, cccDNA, hepatocyte, NTCP, HBV, cell culture

## Abstract

More than 350 million people worldwide have been persistently infected with the hepatitis B virus (HBV). Chronic HBV infection could advance toward liver cirrhosis and hepatocellular carcinoma. The intervention with prophylactic vaccine and conventional treatment could suppress HBV, but could not completely eradicate it. The major obstacle for investigating curative antiviral drugs are the incompetence of hepatocyte models that should have closely imitated natural human infection. Here, we demonstrated that an immortalized hepatocyte-like cell line (imHC) could accommodate for over 30 days the entire life cycle of HBV prepared from either established cultured cells or clinically-derived fresh isolates. Normally, imHCs had intact interferon signaling with anti-viral action. Infected imHCs responded to treatments with direct-acting antiviral drugs (DAAs) and interferons (IFNs) by diminishing HBV DNA, the covalently closed circular DNA (cccDNA) surface antigen of HBV (HBsAg, aka the Australia antigen) and the hepatitis B viral protein (HBeAg). Notably, we could observe and quantify HBV spreading from infected cells to naïve cells using an imHC co-culture model. In summary, this study constructed a convenient HBV culture model that allows the screening for novel anti-HBV agents with versatile targets, either HBV entry, replication or cccDNA formation. Combinations of agents aiming at different targets should achieve a complete HBV eradication.

## 1. Introduction

Hepatitis B virus (HBV) infection is a worldwide threat with more than 2 billion infected individuals that would give rise to 350 million chronic HBV carriers. Chronic HBV infection risks the development of liver cirrhosis and hepatocellular carcinoma with approximately 620,000 HBV-related deaths annually [1,2].

Current approved treatment comprises only nucleoside analogs (NAs) and interferon-α (IFN-α). These treatments significantly reduced the HBV viral load in patients. NAs, including entecavir (ETV), lamivudine, telbivudine and tenofovir, suppress HBV replication by targeting viral reverse transcriptase. IFN-α indirectly suppresses HBV through modulating the host immune response and directly interferes with HBV replication in hepatocytes [3,4]. Although IFN-α treatment could be effective in certain circumstances, the viral response rate remains unsatisfied, with unbearable side effects [5,6]. Either NA, IFN-α or PegIFN-α cannot completely eliminate viral infection. Therefore, chronic HBV infection requires life-long supportive treatment with a possibility of ensuing drug resistance.

HBV is a member of the *hepadnaviridae* family belonging to the genus Orthohepadnavirus. Viral particles are composed of partially double-stranded 3.2 kb genomic DNA or relaxed circular DNA (rcDNA) [7]. HBV entry relies on the bile acid transporter, sodium taurocholate cotransporting polypeptide (NTCP) found particularly in hepatocytes [8,9]. Binding of HBV surface antigen (HBsAg) with NTCP triggers a viral entry via NTCP mediated-endocytosis [10]. After releasing from the nucleocapsid, HBV rcDNA is transported into the nucleus. The rcDNA contains various DNA lesions that solicit host cell DNA repair machinery to construct a stable HBV DNA structure, called covalently closed circular DNA (cccDNA) [11,12]. HBV cccDNA is a chromatin-like structure or mini chromosome that serves as the template for all HBV transcripts that would be translated as envelope (S, M and L), core antigen (HBVcAg) and viral polymerase. The HBV transcript also serves as pre-genomic RNA (pgRNA). The 3.5 kb pgRNA is encapsidated and reversed transcribed into rcDNA. Capsids contained rcDNA are then either enclosed with envelope and released from infected hepatocytes as progeny virions, or they are returned to nuclease for conserving cccDNA pool replication [9,10].

The persistency of cccDNA in hepatocyte initiates chronic HBV infection with ensuing severe liver diseases (i.e., cirrhosis and hepatocellular carcinoma (HCC)). To prevent HCC, targeting cccDNA for silencing is necessary to completely eradicate chronic HBV infection [13]. However, the understanding of how cccDNA is formed, transcribed and maintained is still unclear [14]. Contemporary treatment of chronic HBV infection relies on NAs and IFN-α that could at best lessen the viral load. The proposed efficacious therapeutic agent targeting the upstream cccDNA has not yet been developed [15]. Curative therapies has been hindered by the availability of infectible hosts, either a hepatocyte culture or animal model that mimics the chronic phase of infection [16]. HBV infection is limited to hepatocytes with narrow host species. The productive infection takes place exclusively in chimpanzee and human hepatocytes that challenges the study of chronic infection and the cccDNA elimination model [17,18].

The establishment of hepatocyte cell lines that could host HBV started after the identification of NTCP or the solute carrier family 10 member 1 (SLC10A1, a sodium/bile acid cotransporter) as a functional receptor for HBV and HDV [19,20]. Especially, increasing human NTCP level through ectopic expression rendered various hepatocyte cell lines to allow HBV infection [19,21]. Stable transfection of the plasmid-encoding HBV genome into HepG2.2.15, a hepatoma cell line, allowed HBV production with certain features of the HBV life cycle [22,23]. However, the HBV plasmid system could not fully imitate natural HBV infection. Some essential steps (i.e., viral entry and cccDNA formation) were not achieved. The contemporary HBV infection model was established on HepaRG, HepG2.2.15, HepG2-NTCP and HepAD38 as host cells. Although these hepatoma cell lines offered feasibility and reproducibility, they carried abnormal proliferation/gene regulation and a deficit in interferon signaling that made them far from perfect for HBV study [24]. Primary human hepatocytes (PHHs) have been regarded as the gold standard for this HBV infection model [25].

PHHs from both fetal and adult sources could host HBV infection [26,27]. However, PHHs carried several limitations, e.g., short life span, dedifferentiation, rapid loss of hepatic functions, poor viability and batch to bath variations [28]. The application of PHHs for chronic HBV infection or other long-term host-pathogen studies, such as malarial, HCV and dengue infections, are not feasible. Therefore, the investigation for HBV and hepatocyte interaction was limited to a few days after infection, representing only acute and subacute infections. It is not suitable for any chronic HBV infection and cccDNA stability assay. To circumvent this limitation, the micropatterned co-culture (MPCC) model, in which PHHs were co-cultured with supportive stroma cells (3T3-J2), extended hepatic functions up to 4–6 weeks [29], supporting HCV [30], HBV [24], and malarial studies [31]. However, this MPCC model required extensive steps, and suffered batch to bath variations of PHH preparation.

Recently, human hepatocyte-like cells (HLCs), derived from either human embryonic stem cells (hES) or induced pluripotent stem cells (iPSCs), had obtained more attention owing to their applications in regenerative medicine, drug biotransformation and in vitro pathogenic infections [32,33,34]. Several investigators have reported the applications of HLC for HBV maintenance [2,35,36,37]. HLCs could serve as precursor cells for hepatocytes due to the unlimited proliferation potential of ES and iPS cells. However, hepatocytes derived from pluripotent stem cells carried mixed populations of mature and fetal hepatocytes that expressed α-fetoprotein with a minimal level of NTCP protein [34]. HBV infection and cccDNA formation required mature hepatocyte in a quiescent state to maintain cccDNA stability [38]. During HLC proliferation, cccDNA would be degraded and therefore immature HLCs could not sustain long-term HBV infection. We previously developed immortalized hepatocyte-like cells (imHCs) derived from human mesenchymal stem cells (hMSCs) [39]. These imHCs maintained the production of hepatocyte-related markers, such as albumin (ALB), α-fetoprotein (AFP), urea, glycogen, tyrosine aminotransferase (TAT), hepatocyte nuclear factor-4α (HNF-4α), glucose-6-phosphase dehydrogenase, NTCP and all major cytochrome P450 that closely mimicked those of primary human hepatocytes. Moreover, these imHCs could host the human malarial parasite in liver-stage (*Plasmodium vivax*) [40] and dengue virus infection [41].

In these studies, we evaluated the potential of imHCs for their hosting of wild-type HBV infection up to 28 d. This infection model is required for drug screening. This condition entails viral replication, cccDNA stability, viral spreading and the cellular response involving interferon-stimulated genes (ISGs). The imHC was effectively infected by HBV derived from both HBVcc and clinical isolates that allowed the entire HBV life cycle, starting from viral entry to virion release. HBV particles produced from this model can infect naïve hepatocytes, spread to lateral cells and establish a 2^nd^ generation HBV progeny. The treatment of HBV-infected imHCs with nucleot(s)ide analogs or antiviral cytokines decreased the HBV viral load, HBsAg, HBeAg and cccDNA levels. Moreover, the incubation of cccDNA positive imHC with interferon-γ (IFN-γ) significantly diminished the cccDNA pool in hepatocytes. These findings strongly indicated that imHCs would be an excellent platform to study the HBV persistency of clinical isolates and for efficacy evaluations of host-targeting antivirals.

## 2. Materials and Methods

### 2.1. Cell Culture

An immortalized hepatocyte-like cell line (imHC) [39,40], HepG2 cell line (ATCC, Manassas, VA, USA) and HepaRG (Thermo Fisher Scientific, Waltham, MA, USA) were used. All cell lines were cultured in DMEM/F12 (Hyclone), 10% FBS, 100 U/mL penicillin, 100 µg/mL streptomycin at 37 °C, 5% CO_2_. Prior to hepatitis B virus (HBV) infection, 1 × 10^6^ cells were seeded onto each well of a 6-well plate overnight. The quiescent stage was achieved in differentiated medium [42] (Williams’ E medium, 10% FBS, 100 U/mL penicillin, 100 µg/mL streptomycin, 5 µg/mL insulin, 50 µM hydrocotisone, 2 mM L-glutamine (GlutaMax, Gibco, Thermo Fisher Scientific, Waltham, MA, USA) and 2% DMSO (Sigma Corporation of American, Ronkonkoma, NY, USA) for 2 weeks.

### 2.2. Detection of Sodium Taurocholate Cotransporting Polypeptide (NTCP) in Hepatocytes

For mRNA expression, total RNA was extracted by illustra RNAspin Mini RNA isolation kits (GE Healthcare, Chicago, IL, USA). The total RNA (2 µg) was immediately converted to cDNA using ImProm-II™ Reverse Transcription System (Promega, Fitchburg, WI, USA) with the specified primer pairs (Table S1). The qPCR was carried out by KAPA SYBR^®^ FAST qPCR Kits (Kapa Biosystems, Wilmington, MA, USA) at 95 °C for 3 min; 40 cycles of 95 °C for 10 s, 60 °C for 20 s, using Mx3000P QPCR System (Agilent, Santa Clara, CA, USA). For protein analysis, total protein was extracted with RIPA buffer with protease inhibitor. Samples were separated on 10% (*w*/*v*) polyacrylamide gels and transferred to a PVDF membrane. The membrane was incubated with anti-sodium taurocholate cotransporting polypeptide (anti-NTCP) (1:100 dilution, ab131084, Abcam, Cambridge, UK) followed by HRP-conjugated goat anti-rabbit antibody (1:10,000), and detected by Luminata crescendo Western HRP substrate (Millipore, Burlington, MA, USA) and visualized under Omega Lum™ G Imaging System (Aplegen, San Francisco, CA, USA). For loading control, the blot was stripped and probed with mouse anti-GAPDH antibody (AM4300; 1:200,000 dilution) and HRP goat anti-mouse secondary antibody (1:10,000).

### 2.3. Production of HBV from Cultured Cells

Cell culture based hepatitis B virus (HBVcc subtype adw2) was prepared from freshly collected supernatants of the HBV stably transfected HepG2 cell line, clone 2.2.15 [22]. HepG2.2.15 cells were maintained in DMEM (Hyclone, GE Healthcare), 10% FBS, 100 U/mL penicillin, 100 µg/mL streptomycin. The supernatant was collected at 7 d after adding 380 µg/mL G418 and concentrated 100-fold. The collected supernatants were filtered through 0.45 µm and viral particles were concentrated by Lenti-X™ Concentrator (Clonetech, Takara Bio, Mountain View, CA, USA). The mixtures were incubated at 4 °C at least 30 min before centrifugation at 1500× *g* for 45 min at 4 °C following the manufacturer’s instructions. The supernatants were removed and the pellet was gently resuspended with FBS at dilution 1:100 of original volume of supernatant. 100× HBV was aliquoted and stored at −80 °C until use. The HBV titer was about 6.28 × 10^8^ HBV genome equivalents/mL using the serial dilutions of a known amount of plasmid HBV 1.3-mer WT replicon (Addgene plasmid # 65459) as a standard curve.

### 2.4. The Infection to Hepatocytes Using HBV Derived from HepG2.2.15 or Clinical Isolate

HBV positive plasma from patients (>10^6^ IU/mL HBV genotype C, 50 µL) was added to host cells in 1 mL Williams’ E serum-free medium, 4% PEG 8000 (89510, Sigma, St. Louis, MO, USA) for 24 h at 37 °C. For HBVcc infection, 10 µL of 10^8^ HBV genome equivalents was added at MOI 100. At the end of incubation, infected cells were vigorously washed thrice with PBS before being cultured in complete Williams’ E medium without DMSO.

### 2.5. Evaluating HBV Progeny Produced from Infected imHC and HepaRG

Differentiated HepaRG and imHC were infected with HBV derived from HepG2.2.15 for 7 d. The conditioned medium was centrifuged and passed through a 0.45 µm syringe filter to remove cell debris. PEG 8000 (8%) in Williams’ E media (0.5 mL) was added to the differentiated naïve HepaRG and imHC. The naïve hepatocytes were infected with 0.5 mL of 2 × 10^5^ IU/mL HBV supernatants for 24 h at 37 °C on 6-well plates. HBV-infected cells were vigorously washed thrice with PBS and maintained in complete Williams’ E medium without DMSO. Conditioned medium was collected and renewed on day 3. Cells were harvested on day 7 post-infection. Cell pellets and supernatants from a secondary HBV infection were further quantified for HBV DNA.

### 2.6. Measuring the HBsAg and HBeAg in Supernatant Using ELISA

Differentiated HepaRG and imHCs were cultured on 6-well plate until confluence. Both cell lines were infected with HBV derived from HepG2.2.15 or HBV^+^ plasma. The inoculum was removed followed by vigorous washing, and conditioned medium was harvested every 3 d during days 7–28 post-infection. Hepatitis B surface antigen (HBsAg) and hepatitis B e antigen (HBeAg) were measured using an ELISA kit (KA0286 and KA0290, Abnova, Taiwan). The quantitative analysis of HBsAg and HBeAg were calculated using a standard curve plotted from HBV recombinant proteins.

### 2.7. Immunofluorescent Staining

The imHCs were cultured onto 96-well CellCarrier-96 optic black plates (PerkinElmer, Waltham, MA, USA) and stained with antibodies against hepatocyte markers: Albumin (ALB) (1:100 dilution, ab10241, Abcam), α-fetoprotein (AFP) (1:100 dilution, SC8399, Santa Cruz Biotech, Dallas, TX, USA), LDLR (1:100 dilution, SC373830, Santa Cruz Biotechnology), sodium taurocholate cotransporting polypeptide (NTCP) (1:100 dilution, ab131084, Abcam), MRP2 (1:100, AB3373, Abcam) and hepatocyte nuclear factor-4α (HNF-4α) (1:100 dilution, SC6556, Santa Cruz Biotech). For detecting HBV infectivity, infected hepatocytes were stained with antibodies against HBV proteins: HBcAg (1:100 dilution, ab8637, Abcam), HBsAg (1:100 dilution, ab20758, Abcam). Hepatocytes were then incubated with goat anti-mouse Alexa Fluor^®^ 488-conjugated (1:500 dilution, Invitrogen, Thermo Fisher Scientific, Waltham, MA, USA), goat anti-rabbit Alexa Fluor^®^ 488-conjugated (1:500 dilution, Invitrogen), or donkey anti-goat Cy3-conjugated secondary antibody (1:500 dilution, BioLegend, San Diego, CA, USA). Hepatocyte nuclei were stained with 2 µM Hoechst 33342 (Thermo Fisher Scientific, MA). Mouse IgG2a, mouse IgG1, rabbit IgG and goat IgG were used as negative control for staining. Fluorescence images were captured by an Operetta High-Content Imaging System (PerkinElmer, MA) with a 40× objective lens.

### 2.8. Detection of Intracellular HBV DNA and Viral Load Using Quantitative Real-Time PCR

Intracellular HBV DNA was extracted from infected hepatocytes using NucleoSpin tissue DNA extraction kit (MN, Düren, Germany). The HBV viral load was measured in conditioned medium. HBV DNA was extracted from 200 µL of conditioned medium using a NucleoSpin blood DNA extraction kit (MN, Düren, Germany). The HBV-specific primers were designed using Vector NTI version 11.5 (Invitrogen, MA). The primer pairs for HBV DNA are 5′-GTTGCCCGTTTGTCCTCTAATTC-3′ and 5′-GGAGGGATACATAGAGGTTCCTTGA-3′. The PCR reaction mix was composed of 50 ng of total DNA, 0.4 µM HBV primers and 10 µL of KAPA SYBR^®^ FAST qPCR Kits (Kapa Biosystems, UK) in 20 µL total volume. HBV DNA was amplified by Mx3000P QPCR System (Agilent Technologies, Santa Clara, CA, USA) with the following condition: 95 °C for 10 s, 60 °C for 20 s and 70 °C for 30 s. The quantitative analysis of the intracellular HBV DNA or viral load was measured by absolute real-time qPCR using HBV plasmid HBV 1.3-mer WT replicon as a calibrator to plot standard curve. HBV 1.3-mer WT replicon was a gift from Wang-Shick Ryu (Addgene plasmid # 65459).

### 2.9. Detection of HBV cccDNA in Infected Hepatocytes Using Quantitative Real-Time PCR

To exclude relaxed circular DNA (rcDNA) from the isolated HBV DNA, covalently closed circular DNA (cccDNA) was extracted from infected hepatocytes using the NucleoSpin plasmid DNA extraction kit (MN, Düren, Germany). The total DNA was incubated with exonuclease to digest contaminating non-cccDNA forms. cccDNA was amplified by specific primers [43] to detect HBV cccDNA with a PCR product of 580-bp fragment, which spans the gap and the nick in the rc form of the HBV genome [44]. The optimized PCR condition consisted of 95 °C for 5 s, 45 cycles at 95 °C for 15 s, 60 °C for 4 s, 72 °C for 25 s and detection at 88 °C for 2 s after each cycle. The specificity to amplify cccDNA over rcDNA appeared to be 10^4^ to 1.

### 2.10. Treatment of HBV Infected Hepatocyte with Entry Inhibitor, Nucleos(t)ide Analogs and Antiviral Cytokines

The naïve imHCs and HepaRG were cultured onto 6-well plates for two weeks in differentiated medium. For HBV entry inhibitor, hepatocytes were pre-treated with 4 µM cyclosporine A for 2 h prior to infection, with HBV particles derived from HBVcc or HBV^+^ plasma. Cyclosporine A was maintained at 4 µM in culture medium for 15 days. For treatment with nucleos(t)ide analogs and antiviral cytokines, imHCs and HepaRG were infected with HBV for 24 h. Infected hepatocyte was treated with 1 µM lamivudine, 1 µM entecavir (ETV), 10 ng/mL TNF-α, 100 IU/mL interferon-α (IFN-α) or 100 IU/mL interferon-γ (IFN-γ) for 15 days. The conditioned medium from infected hepatocytes, mock infection and drug treated group were collected for viral load determination. On day 15, infected imHCs and HepaRG were measured for intracellular HBV DNA.

### 2.11. Determination of Viral Spreading Using Flow Cytometry

Differentiated imHC and HepaRG seeded on 6-well plate were infected with HBV derived from HBVcc or HBV^+^ plasma at MOI 100 in Williams’ E Medium. After 24 h, infected hepatocytes were washed thrice with 1 mL PBS and maintained in Williams’ E Medium, 10% FBS (ES-009-B, Merck, Darmstadt, Germany) for 7 d. Naïve imHCs and HepaRG were stained with 1 µM CellTracker™ Green CMFDA Dye (Thermo Fisher Scientific, MA) for 30 min. For HBV spreading, 5 × 10^5^ infected hepatocytes and 5 × 10^5^ CMFDA-stained naïve hepatocytes were co-cultured on 6 well-plate and maintained in Williams’ E Medium, 10% FBS, 4 µM cyclosporine A and 25 U/mL heparin for 7 d with every other day having a medium change. On day 7, co-culture cells were harvested and fixed with BD perm/wash (BD Biosciences, San Jose, CA, USA) for 20 min. The co-cultured hepatocyte was strained with anti-HBcAg antibody [C1] (ab8637, Abcam, MA) at 4 °C for 30 min, washed thrice with BD wash buffer, stained with Goat anti-Mouse IgG (H + L) secondary antibody conjugated to Alexa fluor 568 at 4 °C for 30 min, and washed thrice with BD wash buffer. The stained cells were analyzed using a BD FACSVerse flow cytometer. The HBV spreading was observed using double positive staining with CMFDA and anti-HBcAg.

### 2.12. Ethics Statement

HBV-positive plasma was collected from consenting patients with chronic HBV infection at Ramathibodi Hospital, Mahidol University. The protocol was approved by the Ethics Committee on Research Involving Human Subjects (2556/250) (date of approval: 06/04/2013). All human subjects were adults > 18 years old, and provided written, informed consent.

### 2.13. Statistical Analysis

All results of experiments were performed in triplicate. Data were expressed as means ± SD, and the statistical analyses were performed using GraphPad Prism 7 software. For comparison between two mean values, a two-tailed unpaired students’ t test was used to calculate statistical significance. One-way Analysis of Variance (ANOVA) was used with Dunnett for multiple comparisons to compare multiple values to a single value or Tukey’s HSD to compare multiple values to each other. A *p*-value less than 0.05 was considered statistically significant.

## 3. Results

### 3.1. Expression of Hepatic Phenotypes and NTCP an Entry Receptor for HBV in imHC

**Figure 1:**
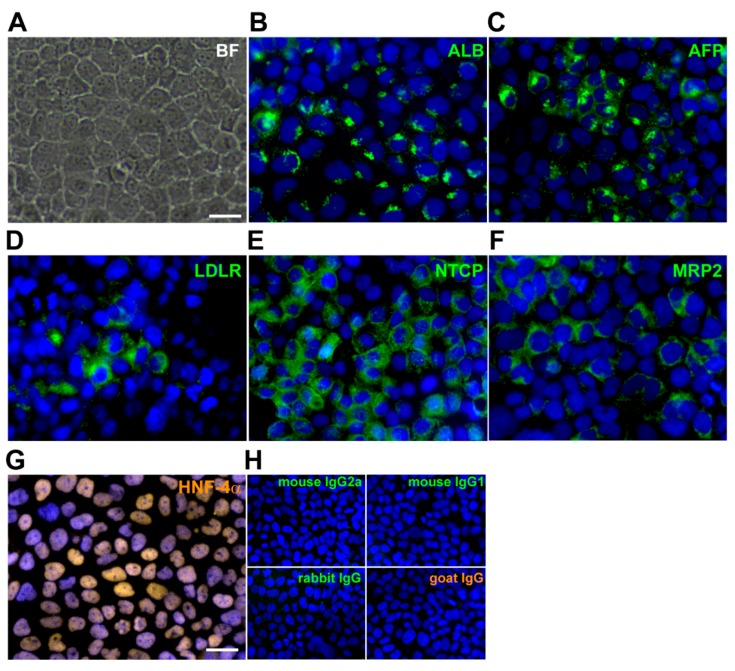
Identification of hepatic markers and the hepatitis B virus (HBV) entry receptor (NTCP) in immortalized human hepatocyte-like cell (imHC). imHCs were maintained in DMEM/F12, 10% fetal bovine serum. After reaching confluence, cells exhibited a usual hepatocyte morphology, including a polygonal shape, binucleated and cord-like structure (**A**). Hepatic phenotypes were examined using immunofluorescence staining for the following major hepatocyte markers: Albumin (ALB) (**B**), α-fetoprotein (AFP) (**C**), low-density lipoprotein receptor (LDLR) (**D**), Na^+^-taurocholate cotransporting polypeptide (NTCP) (**E**), multidrug resistance-associated protein 2 (MRP2) (**F**), hepatocyte nuclear factor-4-alpha (HNF-4α) (**G**) and an isotype control (**H**). Cell nuclei were visualized using Hoechst 33342 DNA dye. Fluorescence images were captured and analyzed using an Operetta High-Content Imaging System (PerkinElmer, Waltham, MA, USA) with a ×40 objective lens. Scale bar = 50 μm. The presence of hepatic marker in imHCs was quantified from 15 randomly selected image fields (total number of analyzed cells > 2000).

The precursor cells of immortalized human hepatocyte (imHC) came from hMSC that had been immortalized with human telomerase reverse transcriptase (hTERT) and Bmi-1 prior to the differentiation [45]. After maturation, imHCs were characterized for (1) hepatic markers; (2) continuous cell line phenotype (population-doubling level, PDL); (3) liver architecture (cord-like structure and polygonal-shaped morphology) (Figure 1A); and (4) basic hepatocyte-specific markers (i.e., ALB, AFP, HNF-4α, LDLR, MRP2) through immunofluorescence staining.

Moreover, the sodium-taurocholate cotransporting polypeptide (NTCP), an entry receptor for HBV, was highly expressed in imHCs (Figure 1E). More than 80% of imHC population contained ALB, MRP2, HNF-4α, and especially 90% of imHC carried NTCP (Figure 1B–G). The expression level of LDLR in imHC was comparable to that of the human hepatocellular carcinoma cell line (HepG2) [40]. The expression of the hepatic maturation marker (HNF-4α) together with the canalicular multi-specific organic anion transporter (MRP2) indicated that the imHCs had fully developed into functional hepatocytes. These evidences confirmed that the hepatic phenotypes of imHCs were comparable to those of primary hepatocytes [39].

### 3.2. Presenting of Hepatic Maturation Features and Upregulating of NTCP Receptor Expression in imHC after Hepatic Maturation Induction

**Figure 2:**
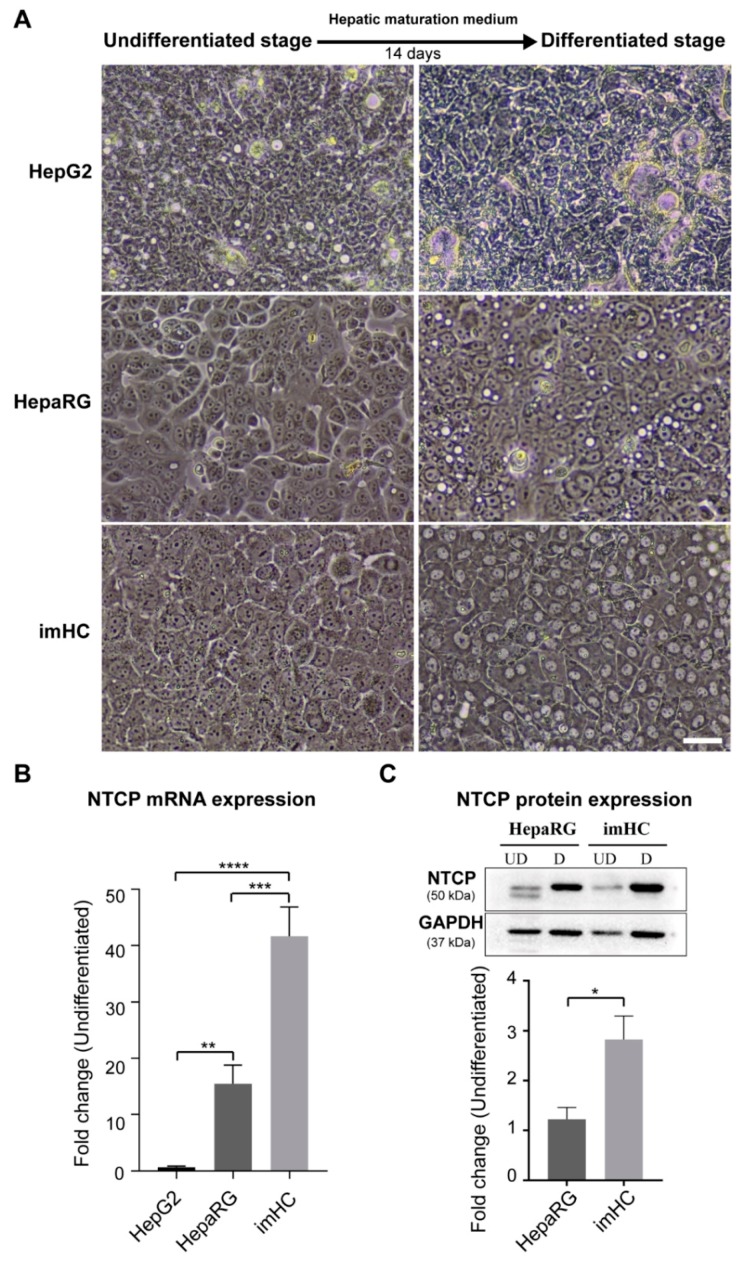
Induction of hepatic maturation of imHC and NTCP characterization in mature cells. The imHC, HepaRG and HepG2 were maintained in hepatic maturation medium for two weeks. Hepatic architectures, such as a cord-like structure, bi-nucleated and polygonal-shaped morphology, signified exclusively the differentiated stage of imHCs (**A**). The scale bars represented 50 µm. After maturation induction, NTCP expression was significantly increased in both imHC and HepaRG (**B**). The level of NTCP protein after normalization with GAPDH was higher in imHC than in HepaRG (**C**). Data are presented as mean ± SD. *, **, *** and **** represented statistical different data with a *p*-value < 0.05, < 0.01, < 0.001 and < 0.0001 respectively.

The imHCs, HepG2 and HepaRG that had been seeded onto a 6-well plate at a density 3 × 10^6^ cell/well in Williams’ medium E with GlutaMAX-I and 10% FBS, exhibited epithelial-like morphology at 100% confluency (Figure 2A). After having been incubated with hepatic maturation medium supplemented with 2% DMSO (*v*/*v*) for 2 weeks, only imHC showed hepatic maturation features, the cord-like structure with bile canaliculi. The imHCs have bi-nucleated cells in the cell population that are usually found in primary human hepatocyte. After exposure to the maturation medium, HepG2 did not exhibit hepatic maturation features, but continued to form multiple layers without contact inhibition. These characteristics typically presented in hepatocytes derived from cancer tissues [46]. Increasing cell density in monolayer was observed in the HepaRG cell. However, the morphology of HepaRG, similar to that of the untreated group, did not progress toward the hepatic maturation feature (Figure 2A). The NTCP, an essential HBV entry receptor, was measured in differentiated hepatocytes. The significant increase in NTCP transcription was detected in HepaRG and imHC at 17-fold and 40-fold, respectively, over that of the untreated conditions. HepG2, after a differentiation attempt, could not drive the NTCP expression (Figure 2B). The Western blot analysis in HepaRG and imHC after maturation induction revealed the increase in NTCP (>50 kDa) by 1.2- and 2.8-folds, respectively (Figure 2C and Figure S1). The upregulation of NTCP after maturation, in agreement with the increasing glycosylated form of NTCP, might confer the susceptibility to HBV entry.

### 3.3. The imHCs were Susceptible to HBV Infection, Sustained Viral DNA Replication and Generated Viral Particles after Infection with either HepG2.2.15 Derived HBV or the Clinical Isolates

**Figure 3:**
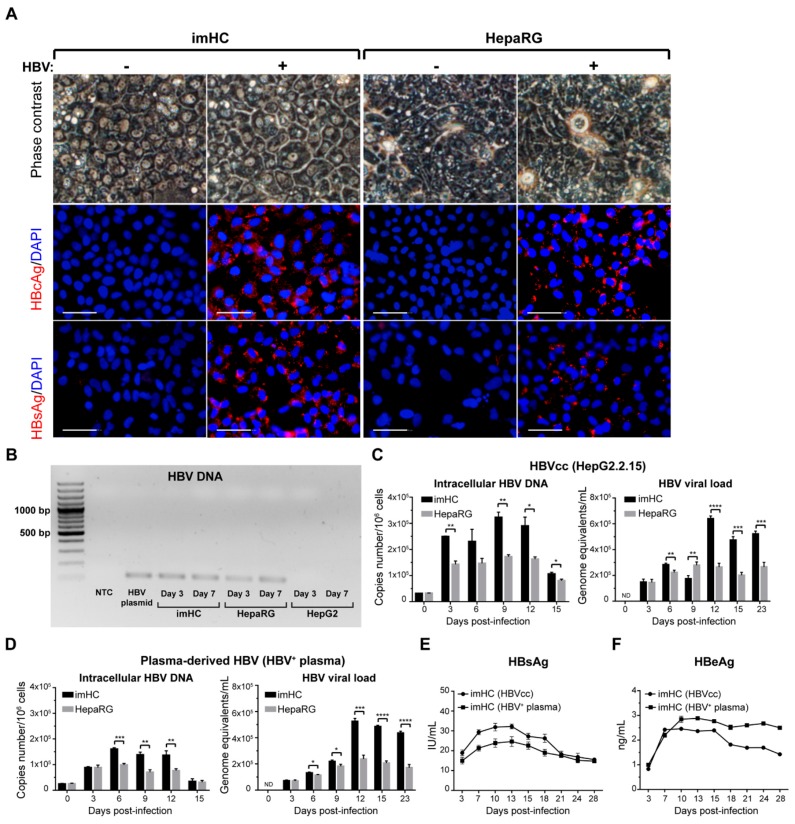
Mature imHCs supported the entire HBV life cycle of both HBVcc and the clinically isolated HBV. The phase contrast microscope with 50-µm scale bars demonstrated the morphology of imHC and HepaRG on day 7 post-infection. The (−) and (+) indicated the mock and HBV infection, respectively. The hepatitis B core antigen (HBcAg) and the hepatitis B surface antigen (HBsAg) were detected only in HBV-infected imHC and HepaRG using an immunofluorescence assay (**A**). The scale bars represented 50 µm. HBV DNA was detected on day 3 and day 7 post-infection in infected imHC and HepaRG, respectively. Infected HepG2, HBV plasmid and NTC were used as infected, positive and negative controls, respectively (**B**). The imHC infected with either HBVcc (**C**) or HBV^+^ plasma (**D**) produced more intracellular HBV DNA and released new HBV particles to the supernatant. HBsAg was highly detected in supernatant on days 7–13 post-infection and persisted until day 28 (**E**). The hepatitis B viral protein (HBeAg) was detected on day 3 post-infection and maintained at 1–2 ng/mL during the experimental period (**F**). Data are presented as mean ± SD. Here, *, **, *** and **** represented statistically different data with a *p*-values < 0.05, < 0.01, < 0.001 and < 0.0001, respectively. ND represented undetectable.

To investigate whether imHCs can host an entire HBV lifecycle, HepaRG and imHC were maintained in maturation medium with 2% DMSO for two weeks prior to the infection. The sources of HBV came from either HepG2.2.15 or the clinical isolate genotype C (HBV^+^ plasma). HepG2.2.15 could continually generate HBV (HBVcc) from its transfected genome. The HBV load of at least 10^6^ IU/mL was selected to infect naïve hepatocytes. Differentiated hepatocytes were infected with HBV 100 genome equivalences per cell (GEQ/cell) in William’s E medium, 10% FBS, 4% PEG 8000. After 24 h of infection, infected cells were vigorously washed five times with 1 mL PBS to remove leftover virus and PEG, and maintained in complete William’s E medium throughout the experiment. The phase contrast microscopic of differentiated imHCs in mock infection (−) or infected (+) with HBV on day 7-post infection did not exhibit CPE or cytotoxicity (Figure 3A). Immunostaining analysis revealed that infected imHCs were positive for the hepatitis B core antigen (HBcAg) and hepatitis B surface antigen (HBsAg) (Figure 3A). Less than imHCs, infected HepaRG population was positive for HBsAg and HBcAg staining. These results indicated that imHCs were superior to the classical hepatocellular carcinoma cell line, HepaRG, as the host cells for HBV (Figure 3A).

The genomic DNA from days 3 and 7 post-infection was extracted using a DNA isolation kit (NucleoSpin Tissue, MN). The total HBV DNA was amplified using the HBV specific primers. The authenticity of PCR products was confirmed through gel electrophoresis. The HBV DNA was initially observed on day 3 with the corresponding expression observed on day 7 in both infected imHCs and HepaRG. These observations correlated with the appearance of HBV proteins in infected hepatocytes. On the contrary, HBV DNA was undetectable in infected HepG2 that indicated ineffective HBV infection (Figure 3B). Quantitative real-time PCR was performed to measure the intracellular HBV DNA and secreted HBV particles. To detect intracellular HBV DNA, total genomic DNA from infected hepatocytes on days 3, 6, 9, 12 and 15 served as templates. To detect HBV viral load, 200 µL of conditioned medium from infected hepatocytes on days 3, 6, 9, 12, 15 and 23 post-infection was extracted for DNA using the NucleoSpin^®^ Blood genomic DNA isolation kit and amplified using HBV specific primers. Intracellular and HBV viral load were calculated using HBV 1.3-mer WT replicon in the HBV standard curve. For HepG2.2.15-derived HBV, intracellular HBV DNA levels on day 3 were 2.5 × 10^5^ and 1.5 × 10^5^ copies/10^6^ cells in imHCs and HepaRG, respectively. On day 6 to day 9 post-infection, the level was raised to 2.6–3.2 × 10^5^ copies/10^6^ cells in imHCs (Figure 3C). In HepaRG, the intracellular HBV DNA was sustained at 1.5 × 10^5^ copies/10^6^ cells during day 6 to day 12. No CPE nor cell detachment was observed in HepaRG and imHCs. The intracellular HBV DNA level in imHCs was maintained at 10^5^ copies/10^6^ cells for more than a month (Figure 3C). For HBVcc infection, the HBV viral load on day 3 after infecting a naïve cell with HBVcc were 1.5 × 10^5^ and 1.4 × 10^5^ copies/mL in imHCs and HepaRG, respectively. The HBV viral load in the supernatant of infected imHC was 3 × 10^5^ copies/mL on day 6, and maintained at 4.3–6.5 × 10^5^ copies/mL until day 23. In comparison, that of infected HepaRG was steadily maintained at 2.5 × 10^5^ copies/mL (Figure 3C).

To examine whether imHCs could host plasma-derived HBV, both imHCs and HepaRG were infected with HBV^+^ plasma. The intracellular HBV DNA levels in imHCs and HepaRG on day 3 were 8.84 × 10^4^ and 8.83 × 10^4^ copies/10^6^ cells, respectively, and increased to 1.4 × 10^5^ copies/10^6^ cells on day 9. These imHCs could maintain HBV DNA at 1.36 × 10^5^ copies/10^6^ cells, while those from HepaRG were 0.76 × 10^5^ copies/10^6^ cells (Figure 3D). HBV viral loads in infected imHCs and HepaRG were 7.1 × 10^4^ and 6.6 × 10^4^ copies /mL, respectively. In imHCs, the viral load was raised from 1.3–5.2 × 10^5^ copies /mL and maintained at 5 × 10^5^ copies/mL until day 23 (Figure 3D). Both infected, differentiated HepaRG and imHCs could sustain the intracellular and HBV viral load at 10^5^ copies/10^6^ cells and 10^5^ copies/ mL over a month. The intracellular HBV DNA in infected HepaRG and imHCs was normalized using GAPDH (Figure S2).

### 3.4. Infected imHCs Released HBsAg and HBeAg into the Surrounding Medium

The start of circulatory HBsAg indicated an acute infection that continued through chronic infection. Circulatory HBeAg indicated active HBV replication and spreading. The intracellular immunostaining of infected imHCs alluded to the production/secretion of HBeAg and HBsAg. To measure the levels of secretory HBsAg and HBeAg, conditioned media (50 µL) from day 3 to day 28 were evaluated with an ELISA kit (Abnova, Taiwan). HBsAg levels were 18.94 and 14.97 IU/mL on day 3 post-infection with HBVcc and HBV^+^ plasma, respectively. The HBsAg level increased to 20–30 IU/mL on day 10–13, and was sustained for several weeks (Figure 3E). The secreted HBeAg was first detected (0.83 and 0.10 ng/mL) on day 3 post-infection with HBVcc and HBV^+^ plasma, respectively. HBeAg levels were increased to 2.40–2.80 ng/mL on days 10–21 and sustained at 1.4–2.5 ng/mL until the end of the experiment (Figure 3F).

### 3.5. imHCs Allowed the Production of Secondary HBV Progeny, cccDNA and Viral DNA Replication

**Figure 4:**
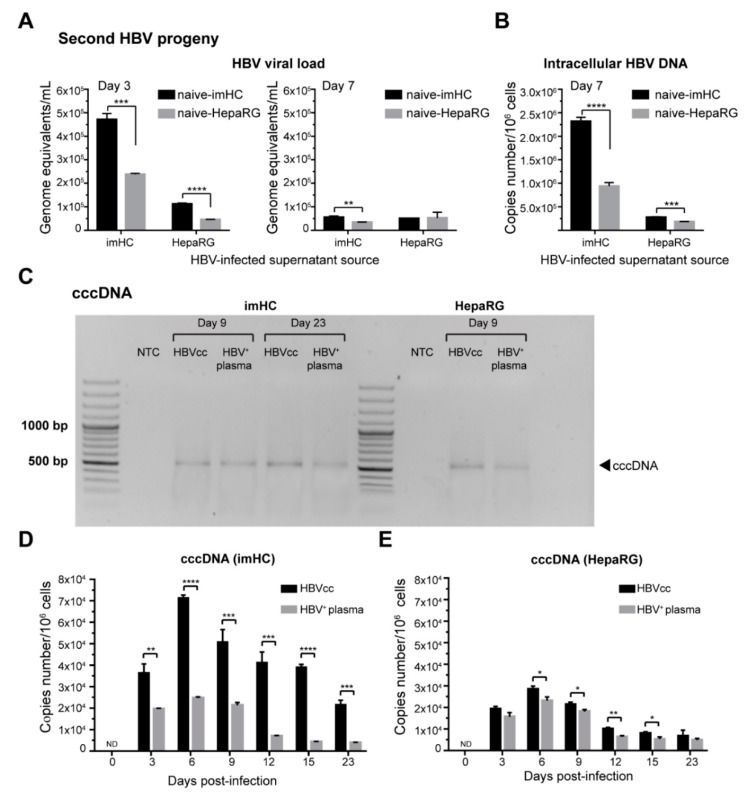
The imHCs infected with either HBVcc or HBV^+^ plasma produced the second HBV progeny and accumulated a covalently closed circular DNA (cccDNA) pool. Naïve imHC and HepaRG were infected with conditioned medium collected from HBVcc infected hepatocytes and monitored for extracellular HBV (viral load) on days 3 and 7 (**A**), as well as intracellular HBV DNA on day 7 (**B**). The cccDNA PCR products were analyzed using agarose gel electrophoresis on day 9 and day 23 post-infection (**C**). The cccDNA pool in either imHCs (**D**) or HepaRG (**E**) after being infected with HBVcc or HBV^+^ plasma was expressed as copies number /10^6^ cells. Data are presented as mean ± SD. *, **, *** and **** represented statistical different data with a *p*-value < 0.05, < 0.01, < 0.001 and < 0.0001, respectively. ND represented undetectable.

To examine whether the host cells infected with the first generation of the HBV particle could yield the second generation of infectious HBV particles, naïve imHC or HepaRG were incubated with the conditioned medium derived from infected imHCs or infected HepaRG. Three days post-infection, the viral loads generated from the infected naïve imHCs and HepaRG were 4.71 × 10^5^ and 2.38 × 10^5^ genome equivalents/mL, respectively (Figure 4A). Changing the HBV source to infected HepaRG yielded the viral loads of 1.11 × 10^5^ and 0.45 × 10^5^ genome equivalents/mL from infected naïve imHC and HepaRG, respectively. On day 7, the viral load from imHC and HepaRG decreased to less than 1 × 10^5^ genome equivalents/mL (Figure 4A). However, the level of intracellular HBV DNA was markedly high (2.32 × 10^6^ copies number/10^6^ cells) in infected naïve imHC with imHC-conditioned medium, while the infected naïve HepaRG condition achieved only 0.94 × 10^6^ copies number/10^6^ cells (Figure 4B). Switching to the conditioned medium from infected HepaRG lessened the intracellular HBV DNA level to below 3 × 10^5^ copies number/10^6^ cells from both imHC and HepaRG (Figure 4B). These results revealed that infected imHC could effectively generate the second generation of HBV particles that promptly infected naïve imHC. The observation of HBcAg positive imHCs after the exposure to the conditioned media of pre-washed infected cells substantiated the production of infectious virions from infected cells (Figure S3).

After binding NTCP, HBV particles fused with the plasma membrane through receptor-mediated endocytosis. The relaxed circular DNA (rcDNA)-containing capsids were released into the cytoplasm that allowed rcDNA to enter the nucleus. To ensure that imHCs supported entire HBV life cycle, infected cells were determined for the presence of HBV cccDNA in infected hepatocytes using a genomic DNA extraction kit. The isolated DNA was treated with exonuclease V to remove linear DNA before being amplified with cccDNA specific primers. Gel electrophoresis on days 6, 9 and 23 post-infection revealed the presence of cccDNA DNA PCR products in imHCs and HepaRG (Figure 4C). The copy numbers of cccDNA on days 3, 6, 9, 12, 15 and 23 were measured using absolute real-time qPCR. On day 3 after infection with either HBV source, cccDNA was detected at 3.64 × 10^4^ and 1.97 × 10^4^ copies/10^6^ cells in imHCs and HepaRG, respectively. The levels of cccDNA increased up to 7.14 and 2.47 × 10^4^ copies/10^5^ cells on day 6; 5.09 × 10^4^ and 2.13 × 10^4^ copies/10^5^ cells on day 9 (Figure 4D,E ). HBV cccDNA in imHCs sustained at 2 × 10^4^ copies/10^5^ cells until day 23. More than 71% of imHCs population carried cccDNA after being infected with HBVcc, while 25% of imHCs population carried cccDNA after HBV^+^ plasma infection. The cccDNA pool in HepaRG varies from 7–27% depending on HBV sources and time post-infection. However, cccDNA in HepaRG was decreased to 0.5 × 10^4^ copies/10^5^ after day 9. HBV cccDNA pool sustained in both infected imHCs and HepaRG, serving as a template for HBV replication over a month of experiment.

### 3.6. Treatment of Infected imHCs with Entry Inhibitor, Nucleos(t)ide Analogs and Antiviral Cytokines Decreased Intracellular HBV DNA and HBV Viral Load

Lessening HBV replication or HBV viral load resulting from the treatment with entry inhibitor, nucleos(t)ide analogs or antiviral cytokines would be a favorable outcome. To verify the applicability of imHCs for HBV drug screening, imHCs and HepaRG were infected with HBVcc or HBV^+^ plasma. One day post-infection, nucleos(t)ide analogs or antiviral cytokines were added to the flesh medium and incubated for 15 d. For entry inhibitor, hepatocytes were pre-treated with 4 µM cyclosporine A for 2 h prior to HBV infection and maintained for 15 d.

**Figure 5:**
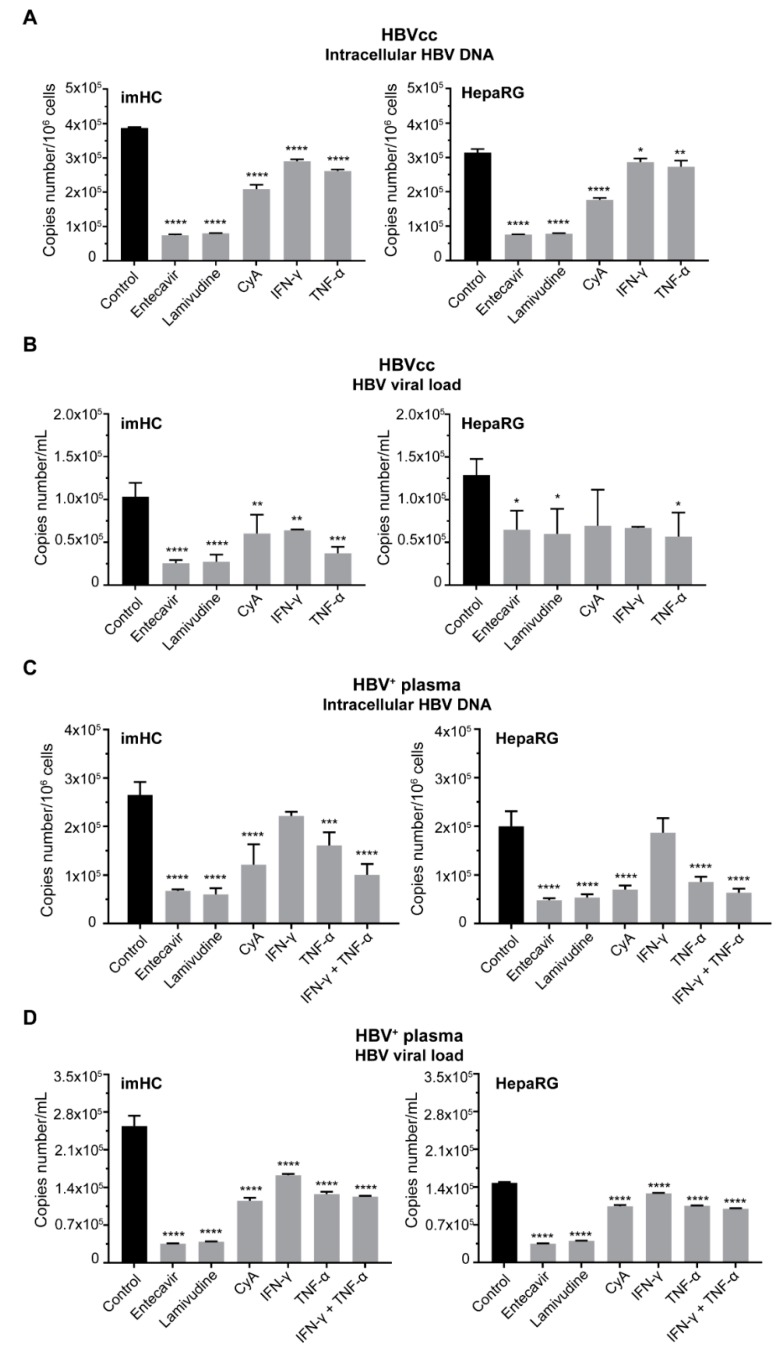
The response to direct-acting antiviral drugs (DAAs) and antiviral cytokines in infected host cells. Both the imHC and HepaRG were infected with either HBVcc (**A**, **B**) or HBV^+^ plasma (**C**, **D**) and treated with DAAs such as entecavir, lamivudine, lamivudine, CyA including antiviral cytokines (IFN-γ, TNF-α). The alteration in intracellular HBV DNA in response to antiviral agents was presented as copies number /10^6^ cells (**A**, **C**). The extracellular HBV (viral load) was measured in conditioned medium using quantitative real-time PCR (**B**, **D**). Infected host cells after being treated with antiviral agents IFN-γ or TNF-α could lessen intracellular HBV DNA derived from HBV^+^ plasma (**C**) and also suppressed HBV viral load in the conditioned medium (**D**). Data are presented as mean ± SD. *, **, *** and **** represented statistical different data with a *p*-value < 0.05, < 0.01, < 0.001 and < 0.0001 respectively.

Entecavir or lamivudine at 1 µM lessened intracellular HBV DNA in HBVcc-infected imHC by 81% or 80%; HBVcc-infected HepaRG by 72% or 74%, respectively. Cyclosporine A decreased intracellular HBV DNA by 54% of in imHC (Figure S4) and 56% in HepaRG. IFN-γ (100 IU/mL) decreased intracellular HBV DNA by 25% in imHC and 8.84% in HepaRG. TNF-α (10 ng /mL) slightly decreased intracellular HBV DNA by 16.62% in imHC and 13.27% in HepaRG (Figure 5A). To evaluate the lessening HBVcc viral load in response to antiviral treatments, infected hepatocytes were treated with antiviral agents for 15 d. HBV DNA was extracted from the conditioned medium. Entecavir, lamivudine, cyclosporine A, IFN-γ and TNF-α decreased the viral load by 75.33%, 73.73%, 41.79%, 38% and 64.15% in imHC; by 49.79%, 53.36%, 73.69%, 48.08% and 56% in HepaRG, respectively (Figure 5B).

For HBV^+^ plasma-infected cells, the treatment with entecavir (1 µM), lamivudine (1 µM), cyclosporine A (4 µM), IFN-γ (100 IU/mL), TNF-α (10 ng /mL), or IFN-γ plus TNF-α decreased intracellular HBV DNA by 74.53%, 77.32%, 54.30%, 16.27%, 39.33%, 61.95% in imHC; 76.11%, 73.25%, 65.11%, 6.46%, 57.12%, 68.21% in HepaRG, respectively (Figure 5C). The reduction viral load derived from HBV^+^ plasma-infected hepatocyte was evaluated after being treated with antiviral agents until day 15. The antiviral agents, such 1 µM entecavir, 1 µM lamivudine, 4 µM cyclosporine A, 100 IU/mL IFN-γ, 10 ng /mL TNF-α and IFN-γ, plus TNF-α, lessened the HBV viral load 86.03%, 84.72%, 54.70%, 36%, 49.83 and 51.58% in imHC, and lessened the HBV viral load 76.47%, 72.77%, 29.34%, 13.08%, 28.87% and 32.36% in the HepaRG supernatant, respectively (Figure 5D). The combination of IFN-γ and TNF-α provided no synergistic effect toward decreasing the viral load (Figure 5D).

### 3.7. IFN-γ Treatment Lessened the HBV cccDNA Level in Infected Hepatocyte

**Figure 6:**
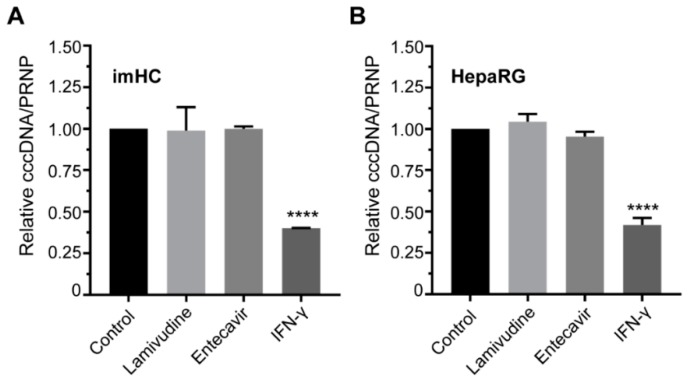

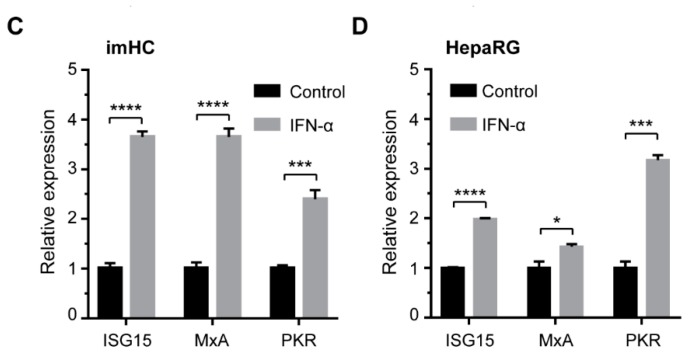
The reduction of HBV cccDNA and the upregulation of antiviral signaling in infected hepatocytes after the treatment with interferon-γ (IFN-γ) and interferon-α (IFN-α), respectively. Mature imHC and HepaRG were infected with HBV and treated with DAAs as well as IFN-γ. The relative cccDNA level in imHC in response to DAAs and IFN-γ was measured and normalized with PRNP in imHC (**A**) and in HepaRG (**B**). The upregulation of antiviral signaling genes (interferon-stimulated gene 15 (ISG15), human myxovirus resistance protein 1 (MxA) and protein kinase R (PKR)) in response to IFN-α treatment were monitored in imHC (**C**) and HepaRG (**D**). Data are represented as mean ± SD. *, **, *** and **** represented statistical different data with a *p*-value < 0.05, < 0.01, < 0.001 and < 0.0001 respectively.

Interferon gamma (IFN-γ) is critical for innate and adaptive immunity against HBV infection by targeting cccDNA. For the cccDNA reduction assay, lamivudine, entecavir or IFN-γ were added to the culture medium on days 3–7 post-infection and the cccDNA level was normalized with PRNP. The quantitative real-time PCR revealed that nucleos(t)ide analogs (lamivudine and entecavir) could not decrease this cccDNA level (Figure 6A,B). IFN-γ decreased the cccDNA level by 60% in imHC and 58% in HepaRG, respectively (Figure 6A,B).

### 3.8. IFN-α Enhanced Antiviral Genes Expression and Innate Immune Response in Infected imHCs

HBV infection could trigger the type I interferon-signaling pathway including interferon-stimulated genes (ISGs). These ISGs exert numerous antiviral effector functions. To investigate the expression of ISGs in imHCs in response to HBV infection and IFN-α treatment, both HepaRG and imHCs were infected with HBV for 7 d and treated with IFN-α on day 3. The expression of interferon-stimulated gene 15 (ISG15), human myxovirus resistance protein 1 (MxA) and protein kinase R (PKR) in response to IFN-α treatment were evaluated in infected hepatocytes. IFN-α increased ISG15 by 1.99 and 3.65 folds; increased MxA by 1.43 and 3.65 folds; increased protein kinase R (PKR) by 3.17 and 2.34 folds in HepaRG and imHCs, respectively (Figure 6C,D).

### 3.9. HBV from Infected imHCs Spread and Secondarily Infected Naïve Hepatocytes in Co-Culture Model

**Figure 7:**
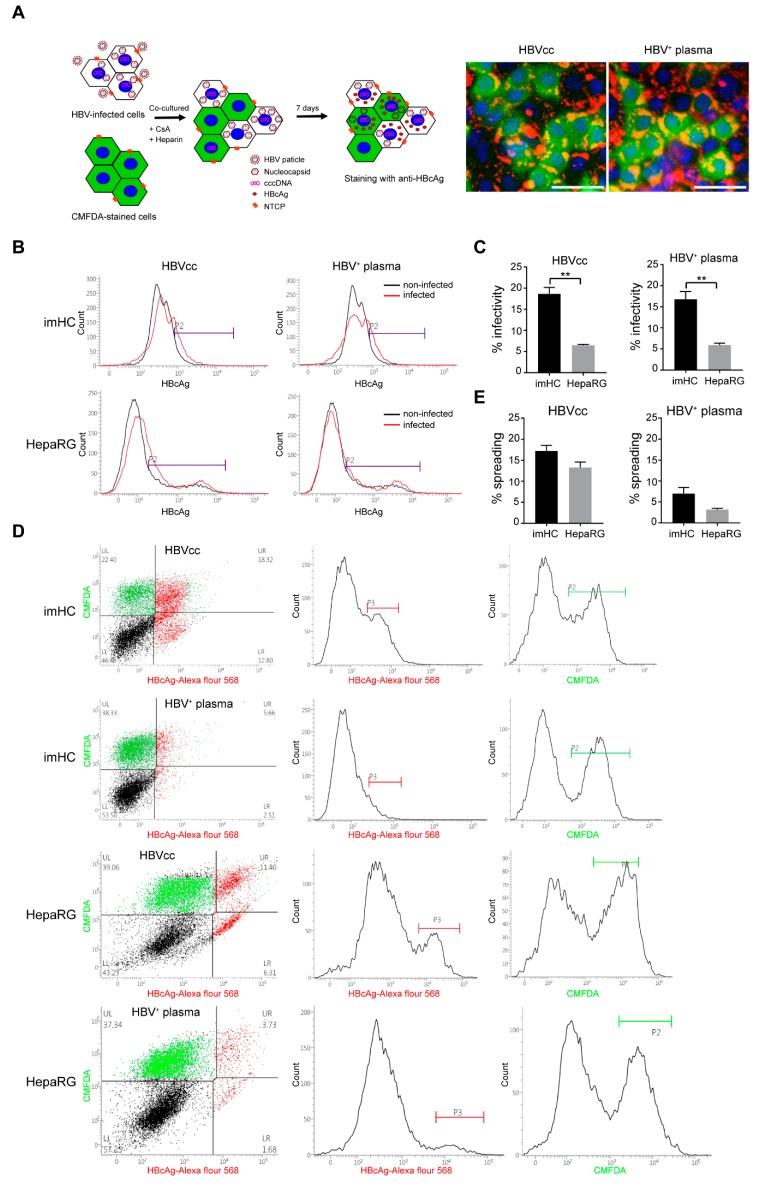
HBV spreading from infected hepatocytes to non-infected cells. Infected hepatocytes were co-cultured with non-infected cells pre-strained with CMFDA. The viral spreading was identified by double positive strained with CMFDA and HBsAg, using a flow cytometer. The diagram of the imHC co-culture model and HBV spreading were illustrated (**A**) with 50 µm scale bars. Population of infected hepatocyte before the co-culture was demonstrated in imHC and HepaRG (**B**) and the percent of infection was analyzed (**C**). Flow cytometry data revealed HBV spreading from infected hepatocytes as CMFDA and HBcAg-Alexa flour 568 double positive cells (**D**). Quantitative analysis of HBV spreading was reported as a percentage from triplicated data (**E**). Data are presented as mean ± SD. *, **, *** and **** represented statistical different data with a *p*-value < 0.05, < 0.01, < 0.001 and < 0.0001, respectively.

HBV enters naïve hepatocytes via NTCP and spreads via intercellular infection. HepaRG and imHCs were examined for hosting lateral HBV infection or spreading. Seven-day post-infection, HepaRG or imHCs were co-cultured with naïve hepatocytes pre-strained with 1 µM CellTracker™ Green CMFDA Dye for 7 d. Naïve and infected cells were treated with 4 µM cyclosporine A to block HBV infection from the supernatant via NTCP. One week after the co-culture, hepatocytes were harvested and strained with anti-HBV core antigen (HBcAg) monoclonal antibody. The stained hepatocytes were analyzed using a flow cytometer and the double positive staining with HBcAg and CMFDA were designated as HBV spreading. The procedure of spreading experiment was illustrated (Figure 7A and Figure S5). The infectivity of HBV in imHCs and HepaRG was evaluated by staining with anti-HBcAg and analyzing by a flow cytometer. The imHCs and HepaRG carrying HBcAg were designated as P2 population (Figure 7B). Infection of imHC and HepaRG with HBVcc yielded 18.64% and 6.48%, infectivity respectively. Changing the HBV source to HBV^+^ plasma (*n* = 3) yielded 16.78% and 5.93% infectivity in imHC and HepaRG, respectively. The HBV infectivity revealed that imHC supported HBV infection approximately 3-fold higher than HepaRG (Figure 7C). To assess the potential of HBV spreading from infected hepatocytes to naïve cells, infected imHC or HepaRG was co-cultured with non-infected CMFDA-strained imHC or HepaRG. After being co-cultured for 7 d, almost 17.25 ± 1.88% of CMFDA-stained imHCs were positive for HBcAg staining. For HepaRG, nearly 13.31 ± 1.81% CMFDA-stained cells were positive for HBcAg staining. Incubating imHC or HepaRG with HBV^+^ plasma and co-cultured with non-infected CMFDA-stained imHC or HepaRG for 7 d, more than 7 ± 1.21% CMFDA-stained imHCs were positive for HBcAg staining. However, at least 3.15 ± 0.46% of CMFDA-stained HepaRG were positive for HBcAg straining 2-fold lesser than that of imHC (Figure 7D). The co-culture of uninfected hepatocytes with non-infected CMFDA-stained cells were used as the negative control, where positivity for HBcAg straining was 0.13% and 0.04% in imHCs and HepaRG, respectively. These results demonstrated that imHCs and HepaRG infected with either HBVcc or HBV^+^ plasma supported viral spreading as demonstrated through the co-culture system.

## 4. Discussion

Study of human hepatotropic pathogens has been hampered in the past due to the shortage of experimental models that sufficiently mimic natural pathogenesis to replace clinical samples or animal models. The first HBV culture model system came from hepatoma cell lines stably transfected with HBV genomic DNA [47]. Another model received the viral genome through adenovirus or baculoviral vectors, followed by productive HBV replication and the production of infectious progeny [48,49]. These models were flawed, due to the lack of viral replication, cccDNA formation, viral entry and other critical steps of the HBV life cycle [18]. The establishment of HepaRG, a hepatoma progenitor cell line, which supports the entire HBV life cycle, offers an essential tool for HBV in vitro studies [50]. However, sustainable HBV infection in HepaRG required at least one month of differentiation prior to infection, and expressed non-glycosylated NTCP protein with karyotype aberrations [51,52].

The primary human hepatocyte (PHH) served as the gold standard for hepatotopic pathogens study for several years [53]. PHH and immortalized PHH were susceptible to HBVcc and patient-derived HBV [26,27,54]. However, the preparation of PHHs from clinical biopsy faced rapid dedifferentiation in culture [55,56,57], triggered by a proliferative response to a pro-inflammatory signal [58]. This compromised their hepatocyte functions and susceptibility to HBV. Proteomic analysis in PHHs revealed lessening in crucial factors for HBV infection, e.g., HNF-4α, RXR and NTCP. The discovery of NTCP as a receptor for HBV entry has paved the way to investigate the complete HBV life cycle using NTCP-expressing hepatocyte cell lines [59,60]. Nevertheless, the transformed hepatoma cells have impaired metabolic activities, intracellular signaling and physiological response [61]. The downregulation of innate immune responses in this hepatoma cell line restricts the study of pathogen–host interactions and diseases progression [62,63]. Using 2D PHH cultures could provide only short-term study, because PHHs are difficult to maintain and rapidly dedifferentiate within several days [55]. The dedifferentiation was minimized in HLCs with sustenance of host factors’ expression for more than a month that favors a long-term HBV infection study.

Recent variations of PHHs still relied upon cell sources with batch to batch variations [64,65,66]. PHHs had limited life-span and rapidly lost HBV infectivity [20,67]. The co-cultures of PHHs with non-parenchymal cells to maintain the hepatic phenotypes were attempted [68,69]. MPCCs model was PHHs donor dependent and required a JAK inhibitor that sustained HBV infection for only 14–19 days [24]. The self-assembling PHHs co-culture (SACC) model extended hepatic function and HBV infection over 30 days, but relied on PHHs sources and the co-culture with the 3T3 J-2 cell line [53,70]. Both MPCCs and SACC PHHs could not support viral spreading to neighboring uninfected hepatocytes [53]. We demonstrated for the first time that imHC infected with fresh clinical isolates allowed lateral spreading of viral progeny. This imHC could effectively serve as a natural host for wild-type HBV and generated infectious viral progeny.

The classical HLCs derived from iPS cells could support HBV infection [35,36] but required 20 days for differentiation and 14 days for maturation, with a low efficiency of HBV infection [2]. We established imHCs [39] that supported the entire HBV life cycle. These imHCs permitted in vitro liver stage development of the human malarial parasite *Plasmodium vivax* [40]. The imHCs were characterized for hepatic markers expression, i.e., albumin (ALB), hepatocyte nuclear factor 4 alpha (HNF-4α), tyrosine aminotransferase (TAT) and drug metabolism enzymes (CYPs).

In this study, imHC could express high levels of ALB, AFP, LDLR, MRP2, HNF-4α and NTCP. After maturation, both mRNA and protein levels of NTCP were significantly increased compared to undifferentiated imHCs. HBcAg, HBV DNA, cccDNA, HBsAg and HBeAg were observed after infection with HBVcc or HBV^+^ plasma, demonstrating the rising of HBV infectivity in imHC. The monitoring of HBV cccDNA level in cells infected with fresh clinical isolate could have made imHC as a robust model to screening for HBV drug sensitivity in clinical practice.

The imHC also established proof-of-work for utilized this model for antiviral drug screening by DAAs and antiviral cytokines. Inhibition of HBV NTCP-mediated entry is examined as a therapeutic strategy to prevent HBV infection, such as after liver transplantation, and could restrict HBV spread in chronically-infected patients. Most current DAAs are effective to reduce intracellular HBV DNA and viral load in HBV-infected imHC. Furthermore, treating infected imHC with IFN-γ reduced 50% of the HBV cccDNA level, while lamivudine and entecavir did not reduce cccDNA. This finding revealed that reduction of cccDNA in imHC could be effected by activation-induced cytidine deaminase (AID) deamination activity [71,72], which activates the lymphotoxin-β receptor via IFN-γ signaling [73]. We also discovered the increasing of interferon-inducible antiviral effectors such as ISG15, MxA and PKR genes, increasing 2.5–3.8 folds after HBV infection and treated with IFN-α. The upregulating of ISG15, MxA and PKR during HBV infection revealed the effective of innate immune responses in imHC.

The effective and rapid innate immune response to pathogens is regulated via IFN (44). The inhibition of IFN activation could induce HBV replication in PHHs [74], and we found HBV-activated IFN response in imHCs comparable to other infected hepatic cells [75,76]. Our results suggest that active IFN signaling could efficiently block viral replication. Moreover, we observed IFN-γ-induced cccDNA degradation in infected imHC. These observations demonstrate that the imHCs sustained innate immune responses similar to the PHHs model.

In theory, an individual HBV particle is adequate for establishing an infection and spreading to all hepatocytes in-patient [77]. Particularly, virus spreading, which appears efficiently in vivo, could not be investigated with the cell culture models [78]. Recent publications identified the HBV spreading from infected hepatocytes to adjacent cells [36]. To investigate whether imHC could support HBV spreading, we stained non-infected imHCs with CMFDA, co-cultured with HBV-infected imHC and lastly stained with anti-HBcAg. The imHCs support HBV transmitted directly from cell to cell or via infection of CMFDA-stained imHCs by an extracellular progeny virus released from infected cells. This result was similar to previous publications using stem cell-derived hepatocyte and HepG2-NTCP [59].

Taken together, using imHCs as an HBV culture model is highly recommended for antiviral screening and testing. In our current study, we were successful to established the hepatocyte model to detect the candidate chemicals which could inhibit both HBV replication and cccDNA elimination, in order to find the complete cure of chronic HBV infection. For instance, targeting NTCP receptors could inhibit viral entry, while targeting HBV polymerase could reduce viral load, and targeting HBV cccDNA could reduce overall HBV markers. Even though single treatment may not achieve HBV eradication, combination of antivirals targeting various steps of HBV life cycle may lead to a complete cure. The imHCs model provides a highly functional hepatocyte system to gain our understanding of virus-host integration including HBV entry, replication and immune response, as well as offering new opportunities for antiviral agent screening and development to achieve a complete cure in the near future.

## Supplementary Materials

The following are available online at https://www.mdpi.com/1999-4915/11/10/952/s1, Figure S1. Western blot analysis of NTCP, Figure S2. The abundance of intracellular HBV DNA after infection, Figure S3. Second HBV progeny could infect naïve imHC, Figure S4. The frequencies of HBcAg positive cells declined after the treatment with CyA, an entry inhibitor, Figure S5. HBV spread from infected hepatocytes to naïve cells, Table S1. Primer sets and conditions used in quantitative real-time PCR.
