# Supplementary material for: An Immortalized Hepatocyte-Like Cell Line (imHC) Accommodated Complete Viral Lifecycle, Viral Persistence Form, cccDNA and Eventual Spreading of a Clinically-Isolated HBV
Source: Viruses. 2019 Oct 16;11(10):952. doi: 10.3390/v11100952 (PMC6832882; doi:10.3390/v11100952)
Supplement: Supplementary file 1 [file viruses-11-00952-s001.zip › viruses-602716-for conversion-supplementary/Table S1.pdf]

Supplementary Material

Figure S1 Western blot analysis of NTCP. The glycosylated (50 kDa) and non-glycosylated-NTCP (32 kDa) were illustrated in the undifferentiated form of both HepaRG and imHC. The differentiation protocol drove NTCP toward the glycosylated form. GAPDH served as the control.

Figure S2 The abundance of intracellular HBV DNA after infection. The host cells (imHC and HepaRG) infected with either HBVcc or HBV^{+} plasma were monitored for the abundance of intracellular HBV DNA. The intracellular HBV DNA levels were normalized with that of GAPDH.

Figure S3 Second HBV progeny could infect naïve imHC. Naïve imHCs were infected with HBV particles derived from imHC (HBVimHC) at MOI of 10 for 7 days. Immunofluorescence was performed to detect HBcAg in infected cells. Naïve imHCs were either mock-infected (A), or inoculated with the conditioned medium containing HBV particles released from infected imHC (B). The infectivity was manually counted from the frequency of HBcAg-positive cell counts per 1000 total cells. The result was 44.36%.

Figure S4 The frequencies of HBcAg positive cells declined after the treatment with CyA, an entry inhibitor. Uninfected imHCs counterstained with DAPI underwent either mock infection (A), or staining with goat-anti mouse-Alexa fluor 568 (isotype control, B). The infected imHCs that have been either untreated (C) or treated with 4 μM CyA (D) were stained with anti-HBcAg-Alexa fluor 568 (D) and counterstained with DAPI.

Figure S5 HBV spread from infected hepatocytes to naïve cells. Infected imHCs were co-cultured with CMFDA-stained naïve cells. The co-culture of uninfected imHCs and CMFDA-stained naïve cells served as a negative control (A). The infected imHCs alone were stained with anti-HBcAg-Alexa fluor 568 (B). The co-culture of CMFDA-stained naïve cells with imHCs infected with either HBV^{+} plasma (C) or HepG2.2.15 (D) exhibited the staining of HBcAg that indicated the presence of “HBV spreading”.

Table S1 Primer sets and conditions used in quantitative real-time PCR.

Table S1. Primer sets and conditions used in quantitative real-time PCR (qPCR)

Gene	Genbank Accession	Sense primer 5'-----> 3' (Tm °C)	Antisense primer 3'-----> 5' (Tm °C)	Amplicon size (bp)	Annealing temp. (°C)	Putative function	References
NTCP	NM_003049.4	GGACATGAACCTCAGCATT GTG (59.8)	ATCATAGATCCCCCTGGAG TAGAT (59.4)	100	60	Na+-taurocholate cotransporting polypeptide	(Appelman et al., 2017)
ISG	NM_005101.4	CACCTGAAGCAGCAAGTGA GCGGGCTGGAG (73.7)	CCGCAGGCGCAGATTCATG AACACGGTGCT (74.1)	150	60	ISG15 ubiquitin like modifier	(Kaneko et al., 2016)
MxA	NM_002462.5	GCCAGCAGCTTCAGAAGGC CATGCTGCAGC (74.8)	GGGCAAGCCGGCGCCGAG CCTGCGTCAGCC (82.1)	150	60	MX dynamin like GTPase 1	(Kaneko et al., 2016)
PKR	NM_0011356 51.3	TTTGAAACATCAAAGTTTT TCACAGACCTA (60.9)	CACAGTCAAGGTCCTTAGT ATTTCAGATGT (62.8)	150	60	protein kinase R	(Kaneko et al., 2016)
HBV DNA	MH981305.1	GTTGCCCGTTTGTCCTCTAA TTC (60.1)	GGAGGGATACATAGAGGTT CCTTGA (60.9)	100	60	HBV polymerase gene	(Untergasser et al., 2006)
cccDNA	MH464854.1	GACTCTCTCGTCCCCTTCTC (58.6)	ATGGTGAGGTGAACAATGC T (57.4)	579	60	HBV genomic DNA	(Untergasser et al., 2006)
PRNP	MH568683.1	AGTCAGTGGAACAAGCCG AG (59.9)	TGGCACTTCCCAGCATGTA G (60.0)	112	60	prion protein	in house design
GAPDH	NG_009349.4	GAAATCCCATCACCATCTT CC (55.0)	AAATGAGCCCCAGCCTTCT C (59.6)	124	60	glyceraldehyde-3-p dehydrogenase	(Sa-Ngiamsuntorn et al., 2016)

References

Appelman, M.D., Chakraborty, A., Protzer, U., McKeating, J.A., and van de Graaf, S.F.J. (2017). N-Glycosylation of the Na+-Taurocholate Cotransporting Polypeptide (NTCP) Determines Its Trafficking and Stability and Is Required for Hepatitis B Virus Infection. *PLOS ONE* 12(1), e0170419. doi: 10.1371/journal.pone.0170419.

Kaneko, S., Kakinuma, S., Asahina, Y., Kamiya, A., Miyoshi, M., Tsunoda, T., et al. (2016). Human induced pluripotent stem cell-derived hepatic cell lines as a new model for host interaction with hepatitis B virus. *Scientific Reports* 6, 29358. doi: 10.1038/srep29358

https://www.nature.com/articles/srep29358#supplementary-information.

Sa-Ngiamsuntorn, K., Wongkajornsilp, A., Phanthong, P., Borwornpinyo, S., Kitiyanant, N., Chantratita, W., et al. (2016). A robust model of natural hepatitis C infection using hepatocyte-like cells derived

from human induced pluripotent stem cells as a long-term host. *Virol J* 13(1)**,** 59. doi: 10.1186/s12985-016-0519-1.

Untergasser, A., Zedler, U., Langenkamp, A., Hosel, M., Quasdorff, M., Esser, K., et al. (2006). Dendritic cells take up viral antigens but do not support the early steps of hepatitis B virus infection. *Hepatology* 43(3)**,** 539-547. doi: 10.1002/hep.21048.
