# Supplementary figures and images for: An Immortalized Hepatocyte-Like Cell Line (imHC) Accommodated Complete Viral Lifecycle, Viral Persistence Form, cccDNA and Eventual Spreading of a Clinically-Isolated HBV
Source: Viruses. 2019 Oct 16;11(10):952. doi: 10.3390/v11100952 (PMC6832882; doi:10.3390/v11100952)

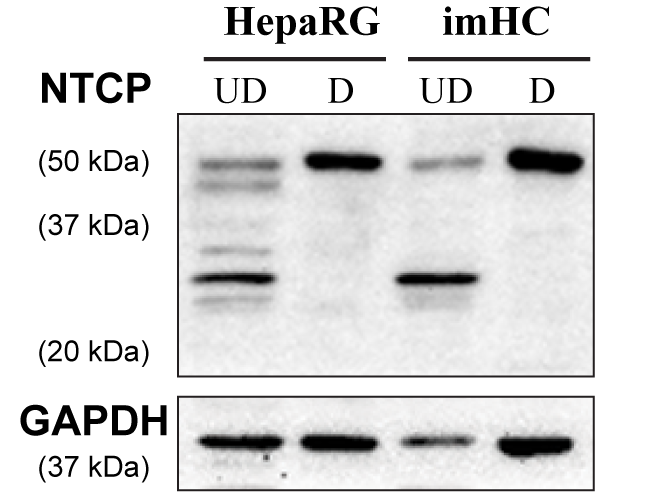

Supplement: Supplementary file 1 [file viruses-11-00952-s001.zip › viruses-602716-for conversion-supplementary/Fig S1.TIF]

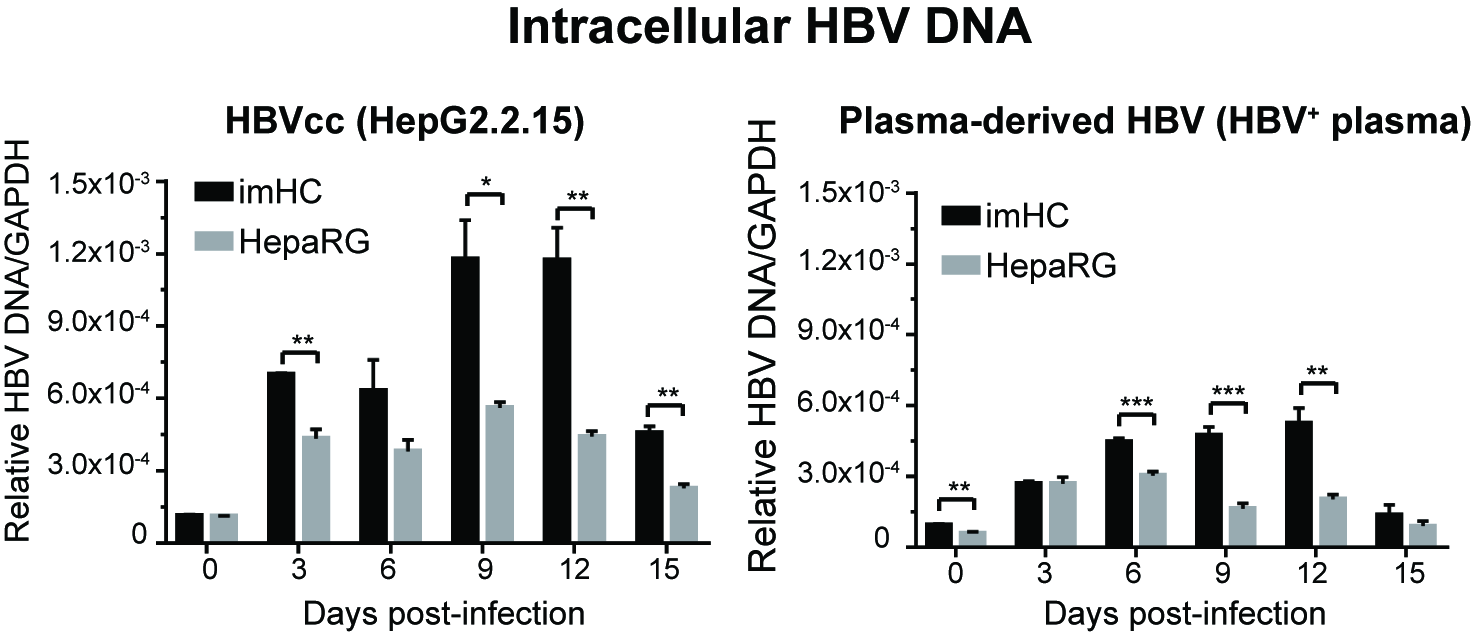

Supplement: Supplementary file 1 [file viruses-11-00952-s001.zip › viruses-602716-for conversion-supplementary/Fig S2.tif]

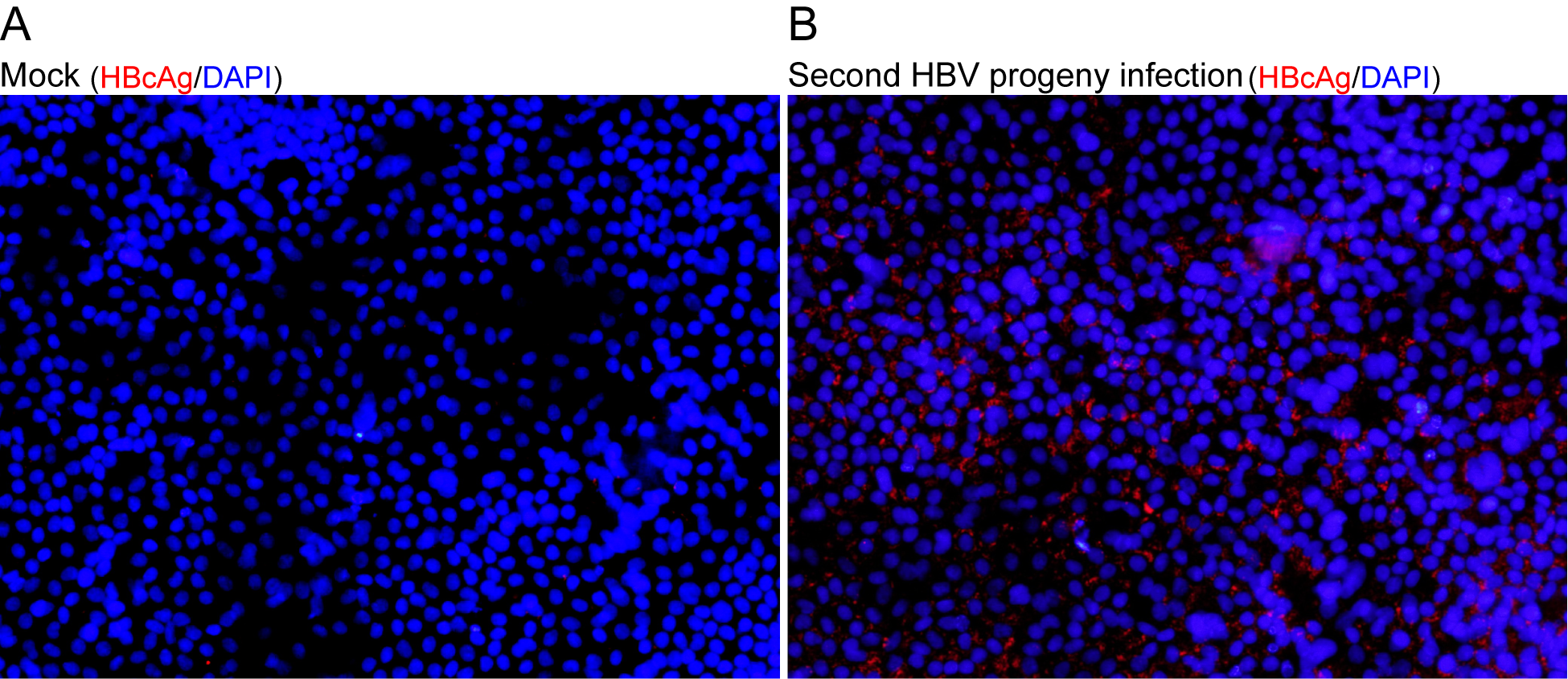

Supplement: Supplementary file 1 [file viruses-11-00952-s001.zip › viruses-602716-for conversion-supplementary/Fig S3.tif]

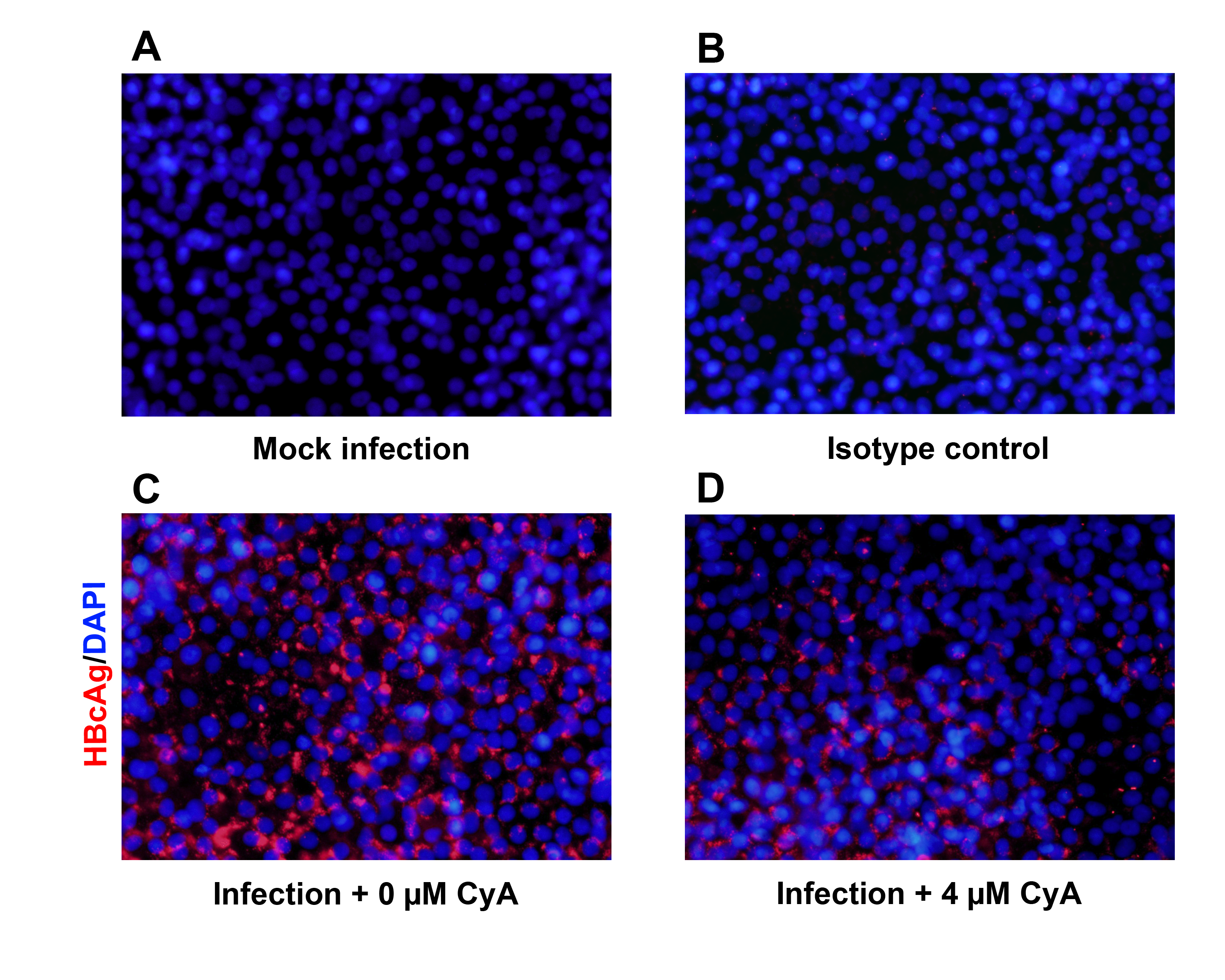

Supplement: Supplementary file 1 [file viruses-11-00952-s001.zip › viruses-602716-for conversion-supplementary/Fig S4.TIF]

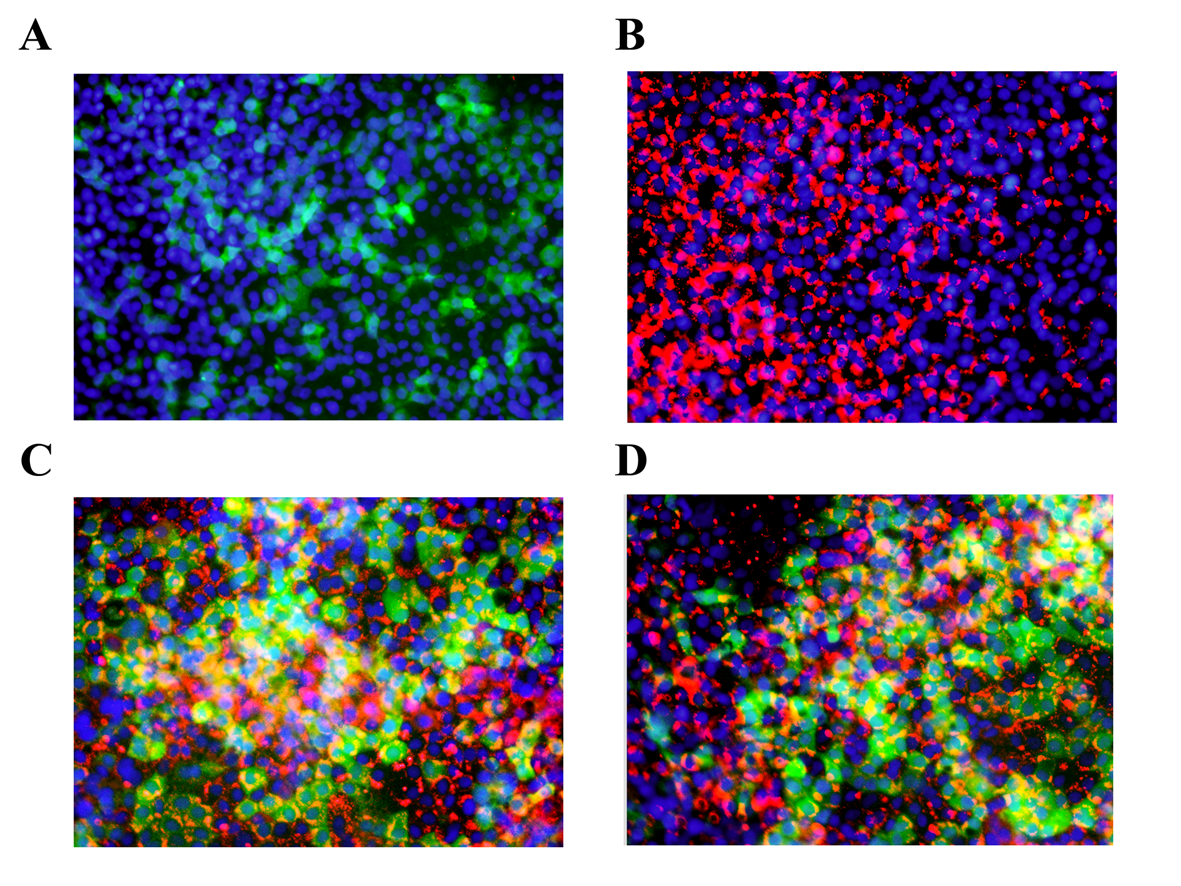

Supplement: Supplementary file 1 [file viruses-11-00952-s001.zip › viruses-602716-for conversion-supplementary/Fig S5.TIF]
